# The temporal and spatial expression pattern of the LGI1 epilepsy predisposition gene during mouse embryonic cranial development

**DOI:** 10.1186/1471-2202-12-43

**Published:** 2011-05-13

**Authors:** Jeane Silva, Guanghu Wang, John K Cowell

**Affiliations:** 1 GHSU Cancer Center, School of Medicine, Georgia Health Sciences University, 1120 15th Street, Augusta 30912, USA; 2 Department of Neurology, Institute of Molecular Medicine & Genetics, School of Medicine, Georgia Health Sciences University, 1120 15th Street, Augusta 30912, USA

## Abstract

**Background:**

Mutations in the LGI1 gene predispose to a rare, hereditary form of temporal epilepsy. Currently, little is known about the temporal and spatial expression pattern of Lgi1 during normal embryogenesis and so to define this more clearly we used a transgenic mouse line that expresses GFP under the control of Lgi1 cis-regulatory elements.

**Results:**

During embryonic brain growth, high levels of Lgi1 expression were found in the surface ectoderm, the neuroepithelium, mesenchymal connective tissue, hippocampus, and sensory organs, such as eye, tongue, and the olfactory bulb. Lgi1 was also found in the cranial nerve nuclei and ganglia, such as vestibular, trigeminal, and dorsal ganglia. Expression of Lgi1 followed an orchestrated pattern during mouse development becoming more subdued in areas of the neocortex of the mid- and hind-brain in early postnatal animals, although high expression levels were retained in the choroid plexus and hippocampus. In late postnatal stages, Lgi1 expression continued to be detected in many areas in the brain including, hippocampus, paraventricular thalamic nuclei, inferior colliculus, and the cerebral aqueduct. We also showed that Lgi1-expressing cells co-express nestin, DCX, and beta-III tubulin suggesting that Lgi1-expressing cells are migratory neuroblasts.

**Conclusion:**

These observations imply that Lgi1 may have a role in establishing normal brain architecture and neuronal functions during brain development suggesting that it may be involved in neurogenesis and neuronal plasticity, which become more specifically defined in the adult animal.

## Background

The Lgi1 gene [1] predisposes to the development of a rare form of partial epilepsy with autosomal dominant inheritance and high penetrance [2], referred to both as Autosomal Dominant Partial Epilepsy with Auditory Features (ADPEAF) [3] and Autosomal Dominant Temporal Lobe Epilepsy (ADTLE) [4]. It has also been shown recently that Lgi1 is the auto antigen responsible for limbic encephalitis, previously thought to be caused by autoantibodies against voltage gated potassium channels [5]. These patients are part of the group of autoimmune synaptic encephalopathies and exhibit seizures and neuropsychiatric disorders. Lgi1 is a secreted protein [6-8] and has also been implicated in cell movement and invasion in glioma cells [9] through suppression of the ERK signaling pathway that results in down regulation of matrix metalloproteinases [10]. Recent studies have shown that Lgi1 interacts with a number of different proteins that are related to synaptic function [11-13], notably members of the ADAM family of proteins (ADAM11, ADAM22 and ADAM23), which represent some of the Lgi1 receptors [12-14]. Most recently [15], Lgi1 was also shown to interact with the Nogo receptor 1 (NgR1) and enhances neuronal growth on myelin based inhibitory substrates and antagonizes myelin-induced growth cone collapse. Mutant null mice for Lgi1 show early onset seizures [16-18], as do mice null for ADAM22 and ADAM23 [19,20]. Thus, Lgi1 represents the only hereditary epilepsy predisposition gene which is not a channelopathy and offers the opportunity to understand different mechanisms behind seizures in these individuals.

Analysis of mutant null mice has demonstrated that Lgi1 is clearly involved in synapse transmission [16,17], although, whether it also plays a role in normal brain development is not clear. Although abnormal structures within the brains of ADLTE patients have been reported by several groups [4,21-23], the level of resolution afforded using current imagining technologies precludes detailed evaluations of these potential abnormalities. Clearly, a better understanding of the cell lineages which express Lgi1 during mammalian brain development will be important in defining its overall function.

Extensive analysis of gene expression in the brain of adult mice demonstrated that the hippocampus and cortex were the predominant regions expressing Lgi1 [24,25], even though the resolution of the cell lineages involved was relatively low using this approach. Immunohistochemical studies in humans have confirmed these broad observations, although many of the antibodies used in these studies have either been shown to cross react with other members of the Lgi1 family of proteins [8] or have not been tested for cross reactivity. To overcome this potential limitation, we developed a BAC transgenic mouse in which the GFP reporter gene was placed under the control of Lgi1 cis-regulatory elements [8]. In these BAC transgenics the GFP gene is expressed in cells which have the transcriptional capability of expressing the endogenous Lgi1 gene, and these cells can be identified using conventional immunohistochemistry. This system has been extensively used as part of the GENSAT program to define expression patterns of genes expressed in the central nervous system of adult mice [26]. To date, however, there have been no reported studies of Lgi1 expression patterns at the cellular level during embryonic development. In this report, we describe an immunohistochemical study of the developing brain and associated structures of the nervous system from embryonic days E9.5-E18.5 and postnatal days P1-P20. These studies show that Lgi1 expression is widespread in the developing brain. More interestingly, co-staining studies shown that Lgi1 is expressed in migratory neuroblast cell lineages.

## Results

### The Lgi1 BAC transgenic system

BAC transgenic technology is now well established [26] and has been used extensively to define gene expression patterns through the insertion of a GFP reporter into the target gene in the context of its endogenous cis-regulatory elements [26]. Thus, with the caveat that regulatory elements that lie some distance away from the promoter may not be present on the BAC, any cell capable of activating the endogenous promoter of a given gene will also activate the reporter gene carried on the transgenic BAC clone. This system has been developed to overcome many shortcomings of antibodies, whose limitations may include, low specificity for the target protein, cross reactivity with homologous proteins, or poor recognition of the endogenous protein in its native conformation. It should be noted, however, that GFP is only a reporter for endogenous gene expression patterns and does not define the intracellular localization of the protein it reports. As such, the intracellular fluorescence signal reflects the ubiquitous distribution of GFP protein. Typically, multiple tandem BAC integrations occur during construction of the transgenic animals which fortuitously provides a strong GFP signal. In our case, >40 copies of the BAC are present in mice used in this study [8]. This reporter system, therefore, has the added advantage of allowing the visualization of expression patterns for genes which normally show low levels of expression or which, like Lgi1 [6,8], are normally rapidly secreted and so may be more difficult to visualize using traditional antibodies. Importantly, since the BAC shows multiple tandem integration events [8], the intensity of the GFP fluorescence does not reflect the intensity of the endogenous gene expression, although relative expression levels between different cells in the same field can be used as a means of determining comparative Lgi1 expression levels.

### Lgi1 expression during embryonic development

**Figure 1:**
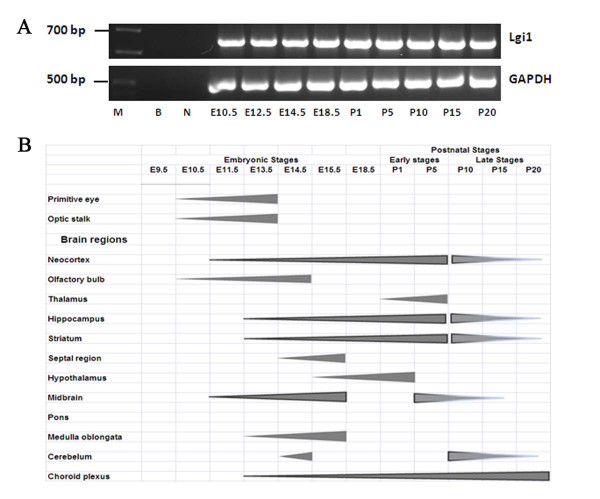
**Lgi1 expression during the developing and the postnatal mouse brain**. **A**: RT-PCR analysis of Lgi1 expression in the brain from embryonic days E10.5 to E18.5 and postnatal cortex P1 to P20. **B**: Summary of the spatial and temporal expression of Lgi1 in mouse sensory organs during brain embryogenesis.

To investigate the distribution of Lgi1 expression in the mouse brain during development, we collected different embryonic/postnatal stage brains from the EGFP-reporter BAC transgenic mice, which we previously showed can be used to define Lgi1 expression patterns at the cellular level [8]. In this analysis we focused on the brain regions that have been implicated in epilepsy, but did not restrict the analysis to the development of specific systems. If Lgi1 influences normal development, then establishing its overall expression pattern during brain development would be important. Most of the sections used in this study were mid-coronal and mid-sagittal, since these planes allow a symmetrical view of the tissue that permits a comprehensive visualization of areas of interest, such as neocortex, hippocampus, nerve ganglia, and sensory organs. Using this strategy, we characterized Lgi1 expression patterns during early development (E9.5-E18.5). A summary of temporal expression patterns in major regions of the brain is given in Figure 1 and specific details at each developmental stage are described below.

**Figure 2:**
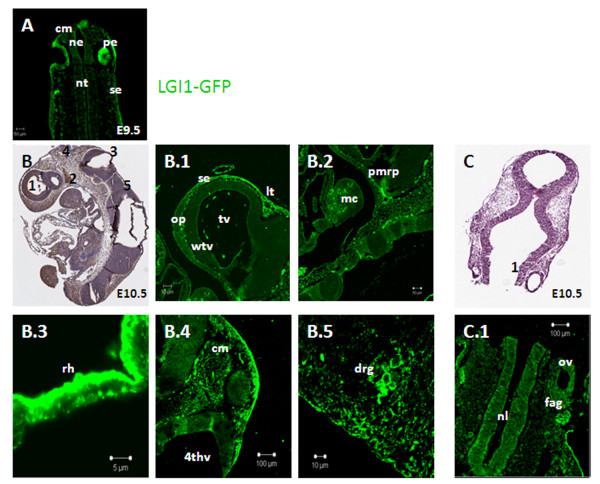
**Photomicrographs showing the expression of Lgi1**. In **A** E9.5 embryos showing a coronal view of the cephalic region with high-level Lgi1 expression in the primitive eye (pe). In **B**, a sagittal view of the E10.5 mouse whole embryo (anti-GFP-peroxidase stained) indicating regions seen in Figures B.1-5 showing the dorsoventral patterning of Lgi1 expression in the brain. The highest levels of Lgi1 expression were seen (B.3) in the roof of the hindbrain (rh), (B.4) mesenchymal connective tissue (cm) and (B5) dorsal root ganglion (drg). In **C**, a frontal section (H & E staining) of an E10.5 mouse brain identifying regions shown in Figure (C.1), identifying Lgi1 expression in the otic vesicle (ov) and facio-acoustic ganglion (fag). Other abbreviations: Skin ectoderm (se), neuroepithelium (ne), neural tube (ne), telencephalic vesicle (tv), wall of telencephalic vesicle (wtv), olfactory placode (op), lamina terminalis (lt), peripheral margin of the Rathke's Pouch (pmrp), mandibular component of the first brachial arch (mc), 4^th ^ventricle (4thv), neural lumen (nl). Scale bars = 100 μm (B.4, C.1), 50 μm (A, B.1, B.2), 10 μm (B.5), 5 μm (B.3).

E9.5 represents the earliest embryonic stage in our analysis, partly because of the technical difficulties associated with processing earlier embryos, and partly because prior to this stage, brain development is still rudimentary. At E9.5, the neural tube is still developing and the structures that will eventually give rise to the brain are largely absent. At this embryonic stage, Lgi1 was prominently expressed in the primitive eye (Figure 2A). Lgi1 was also detected in the skin ectoderm (surface ectoderm), the mesenchymal connective tissue, the optic vesicle, and the neuroepithelium lining the neural tube (Figure 2A). Following neural tube closure at embryonic stage E10.5, Lgi1 expression was found in sensory and secretory organs, such as olfactory placode (Figure 2B.1) and the peripheral margin of Rathke's pouch (Figure 2B.2), which gives rise to the anterior pituitary. Additionally, strong expression could be seen along the roof of the hindbrain (Figure 2B.3) and midbrain mesenchymal connective tissue (Figure 2B.4). Other notable areas of Lgi1 expression at E10.5 included ganglionic tissues, such as facio-acoustic ganglion (Figure 2C.1) and dorsal ganglia (Figure 2B.5). In the forebrain, Lgi1 showed strong expression in the skin ectoderm, lamina terminalis, and the mandibular component of the first branchial arch (Figure 2B.1 and 2B.2), which gives rises the glands of the anterior two thirds of the tongue and ossicles of the middle ear.

**Figure 3:**
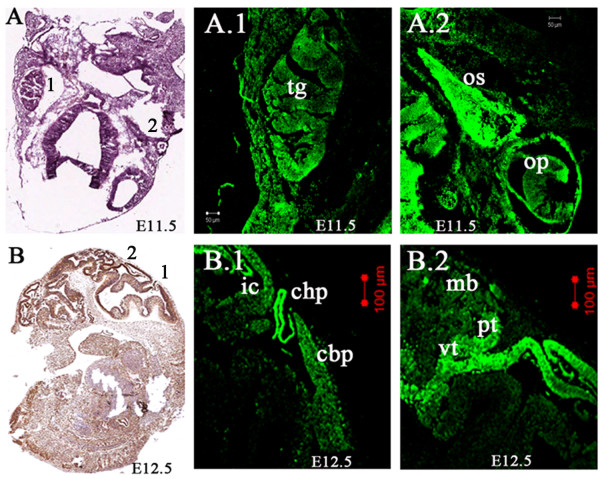
**Lgi1 expression at embryonic stages E11.5 and E12.5**. **A **Coronal section (H & E staining) identifies regions seen in Figures A.1 and A.2. In **A.1**, Lgi1 expression was seen in the trigeminal ganglion (tg), optic stalk (os), and optic cup (op). **B **Sagittal view of the E12.5 mouse whole embryo identifying regions seen in Figures B.1 and B.2. In **B.1**, a midsagittal section (anti-GFP-peroxidase stained) of the cephalic region shows Lgi1 expression in the choroid plexus (chp), inferior colliculi (ic), and cerebellar plate (cbp). In **B.2**, a midsagittal section at the cephalic region of the embryo showing Lgi1 expression in the pretectum (pt), ventral tegmental area (vt) and midbrain (mb). Scale bars = 100 μm (B.1, B.2), 50 μm (A.1, A.2).

Lgi1 expression became notably more extensive throughout the developing brain with increased expression in the forebrain, primarily along the wall of the telencephalic vesicle, although weak expression of Lgi1 was seen in the primitive neocortex. At embryonic stage E11.5, several cranial nerve ganglia showed prominent expression, particularly in the relatively large trigeminal ganglion (Figure 3A.1). In addition, Lgi1 expression was strong in the optic stalk and optic cup (Figure 3A.2). As the brain developed, Lgi1 expression at E12.5 was observed in the roof of the neopallial cortex (future cerebral cortex), choroid plexus, and areas of the midbrain, such as the pretectal area, the anterior ventral tegmentum, tectum, and superior colliculus (Figures 3B.1 and 3B.2). More caudal structures, such as the inferior colliculus and areas of the hindbrain (the pons and cerebellar neuroepithelium) showed moderate levels of Lgi1 expression.

**Figure 4:**
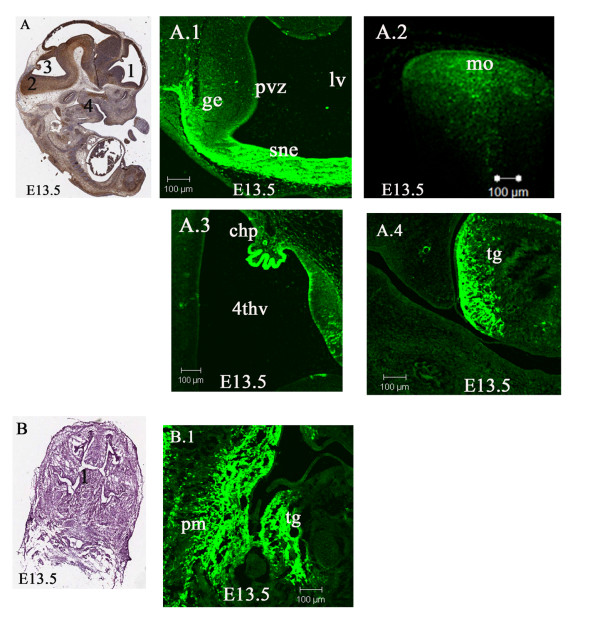
**Lgi1 expression at embryonic stage E13.5**. **A** Sagittal view (anti-GFP-peroxidase stained) of the whole embryo indicating areas of Lgi1 expression seen in Figures A.1 - A.4. **A.1 **Lgi1 expression is seen in cells in the periventricular zone (pvz), ganglionic eminence (ge) and septum neuroepithelium (sne). Lgi1 is also expressed in **A.2** the medulla oblongata (mo) and **A.3** the choroid plexus adjacent to the 4^th ^ventricle (4thv). **A.4 **In the oral cavity, Lgi1 is expressed in the tongue (tg). **B **Frontal section (H & E staining) indicating the area seen in Figure B.1. **B.1 **Frontal section showing Lgi1 expression throughout the tongue (tg), mandibular process and lower molar region (pm). Scale bars = 100 μm.

By embryonic stage E13.5, Lgi1 expression was observed in the roof of the neopallial cortex, areas of the diencephalon and cranial nerve ganglia, such as trigeminal, facio-acoustic, and vagus. Strong expression was found in the periventricular zone, ganglionic eminence (Figure 4A.1), and medulla oblongata (Figure 4A.2). Expression in the ganglionic eminence was now detected for the first time. The ganglionic eminence contains progenitor cells that are destined to become part of the olfactory cortex as well as the striatum [27,28]. Lgi1 was also found to be expressed in several non neural tissues, such as the tongue (Figure 4A.4) and lower molar region (Figure 4B.1) which represents the first signs of ossification. Lgi1 was now strongly expressed in the choroid plexus (Figure 4A.3).

**Figure 5:**
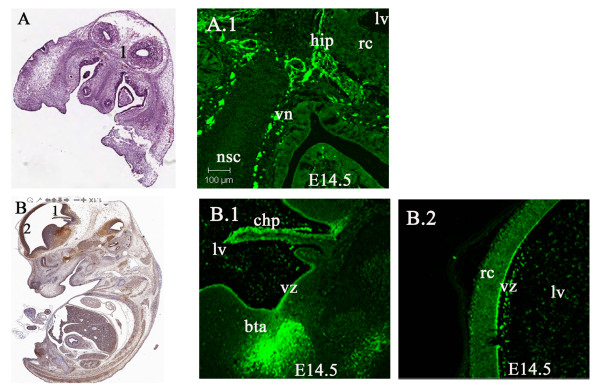
**Frontal and sagittal views of LGI1 expression in the E14.5 mouse brain**. **A** Frontal view (H & E staining) of the head identifying the regions seen in Figure **A.1 **which shows Lgi1 expression in the hippocampus (hip) and vomeronasal nerve (vn). Lgi1 is not detected in nasal septum cartilage (nsc) and shows punctate expression in the roof of the neopallial cortex (rc). **B **gittal view (anti-GFP-peroxidase stained) of whole embryo showing regions of the brain seen in Figures B.1 and B.2. **B.1** Shows Lgi1 expression in the choroid plexus (chp), basal telencephalic plate (bta), and ventricular zone (vz). **B.2 **Shows expression of Lgi1 in the roof of the neopallial cortex (rc) and ventricular zone (vz). Lateral ventricle (lv); Scale bars = 100 μm.

E14.5 Lgi1 expression began to subside in the olfactory lobes by this embryonic stage, as soon as the bulbs became apparent. Lgi1 was also detected in cells of the emerging hippocampus, which appeared as a thin line of cells (Figure 5A) surrounding the cortical neuroepithelium. Within the telencephalon, high levels of expression were found in the basal telencephalic plate, specifically in the posterior region (Figure 5B.1). Cells from this area give rise to the corpus striatum, which becomes part of the basal ganglia. Moderate Lgi1 expression was seen in the cortical neuroepithelium and periventricular zone (Figure 5B.2). The superior sagittal sinus mesenchymal connective tissue and skin ectoderm, as well as the thalamus, was generally devoid of Lgi1 expression at this stage, although several structures immediately superior to the thalamus, including the pretectum, showed moderate levels of Lgi1 expression. Expression in the developing cerebellum at this stage of development was weak. In contrast, strong levels of Lgi1 expression were seen in septal neuroepithelium, cranial nerve ganglia of the vestibulocochlear and vagus, vomeronasal, olfactory and maxillary nerves as well as the choroid plexus. At this point of the mouse development, isolated patches of expression were also seen in non-neuronal organs, such as the cartilage primordium of the vertebra, the tongue and molar tooth, demonstrating that Lgi1 expression is not restricted to neuronal tissues as suggested previously in adult animals [8].

**Figure 6:**
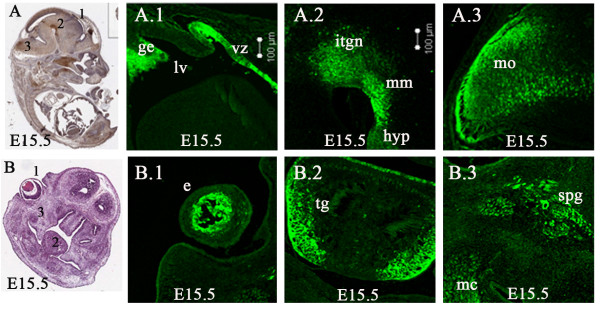
**Lgi1 expression in the E15.5 mouse embryo**. **A** Sagittal view (anti-GFP-peroxidase stained) identifying areas of Lgi1 expression seen in Figure A.1 and A.2. **A.1 **Shows strong Lgi1 expression in the ventricular zone (vz) and ganglionic eminence (ge). **A.2 **Lgi1 was highly expressed in the lower tegmental neuroepithelium (itgn), mammillary body neuroepithelium (mm), and posterior hypothalamus (hyp). **A.3 **Shows the expression pattern through the medulla oblongata (mo). **B **Coronal section (H & E stained) of the head identifying regions seen in B.1-3. **B.1 **Shows Lgi1 expressing cells in the eye (e). **B.2 **Shows Lgi1 expression in the tongue (tg) and **B.3 **shows Lgi1 expressing cells in the sphenopalatine ganglion (spg) and Meckel's cartilage (mc). Lateral ventricle (lv). Scale bars = 100 μm.

By E15.5 (Figure 6), Lgi1 expression patterns largely reflected those observed at E13.5 and E14.5, although as embryonic growth continued, the regions showing Lgi1 expression became more extensive with the increasing size of the brain. Deep within the telencephalon, the ganglionic eminence (Figure 6A.1) continued to show Lgi1 expression. Strong expression was also seen in the ventricular zone (Figure 6A.1). Thalamic cells appear not to express Lgi1, although some areas showed relatively weak expression. Robust Lgi1 expression was found in the lower tegmental neuroepithelium, mammalary body, and posterior hypothalamus neuroepithelium (Figure 6A.2). High levels of Lgi1 expression were now found within the subpial and parenchyma layers of the medulla oblongata (Figure 6A.3). Expression in the eye (Figure 6B.1), tongue (taste buds) (Figure 6B.2), Merckel's cartilage, jaw, and ganglia, as well as in the sphenoid palatine ganglion (Figure 6B.3), remained strong at this stage.

**Figure 7:**
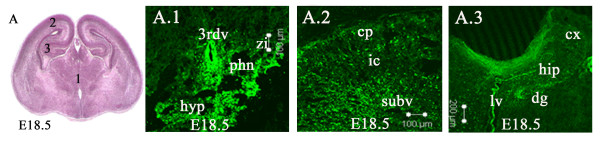
**Coronal views of Lgi1 expression pattern in the E18.5 mouse brain**. **(A)**. Frontal section (H & E stained) indicating areas shown in Figures A.1-3. **A.1**Shows Lgi1 expressing cells in the 3^rd ^ventricle (3^rd^v), paraventricular hypothalamic nucleus (phn), zona incerta (zi) and anterior hypothalamic area (hyp). **A.2**. Shows Lgi1 expression in the cortical plate (cp), intermediate cortical layer (ic), and subventricular zone (subv). **A.3**. Shows Lgi1 expression in dentate gyrus (dg), choroid plexus of lateral ventricle (lv), cortex (cx) and hippocampus (hip). Scale bars = 100 μm (A.1, A.2); 200 μm (A.3).

By embryonic day 18.5 (Figure 7), Lgi1 expression patterns became more defined in specific regions, such as the subventricular zone, hippocampus, choroid plexus, sensory organs, and nerve ganglia. Strong expression was seen in the hypothalamus regions, including the anterior hypothalamic area, paraventricular hypothalamic nucleus, and zona incerta (Figure 7A.1). The cerebral cortex showed low levels of expression, although a thin layer of cells expressing Lgi1 could be seen above the genu of the corpus callosum, which marked the formation of the subplate. Figure 7A.2 shows expression of Lgi1 in the cortical plate, intermediate cortical zone, and subventricular zone. By this time, the hippocampus had also undergone further differentiation, but retains Lgi1 expression, albeit at lower levels, remaining highly expressed in the dentate gyrus (Figure 7A.3). Lgi1 had been weakly expressed in thalamus in the earlier embryonic stages and still appeared weak in the later embryonic stages. Strong expression continues to be seen in the choroid plexus.

### Lgi1 expression in the early postnatal stages of the murine brain

**Figure 8:**
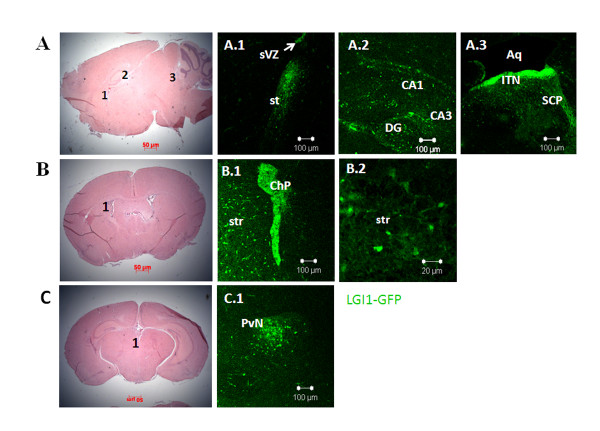
**Coronal and sagittal views of Lgi1 expression in (A) P1 and (B and C) P5 mouse brains**. **A**. Sagittal section of the brain stained with hematoxylin and eosin used as reference to figures A.1-A.3. **A.1**. Shows Lgi1 expressing cells in the subventricular zone (sVZ) and stria terminalis (st) at P1. **A.2**. Shows Lgi1 expression in hippocampus CA1 and CA3 as well as the dentate gyrus (DG) at P1. **A.3**. Shows Lgi1 expression in the inferior tectal neuroepithelium (ITN) and superior cerebellar peduncle (SCP) in the P1 brain. **B**. Coronal section of the brain stained with hematoxylin and eosin as reference to figures B.1 and B.2. **B.1**. shows Lgi1 expression in the choroid plexus (Chp) and striatum (str) at P5. **B.2**. shows Lgi1 positive cells in the striatum (str) in high magnification. **C**. Coronal section of the brain stained with hematoxylin and eosin as reference to figure C.1. **C.1**. illustrates Lgi1 expressing cells in the paraventricular nucleus (PvN) at P5. Aq = Aqueduct. Scale bars = 100 μm (A.1 to A.3, B.1 and C.1); 20 μm (B.2).

At postnatal day one (P1), moderate to low expression of Lgi1 was present in several areas described above (Figure 1). The lowest levels of Lgi1 expression were seen along the neocortex, and were almost undetectable in the frontal and cingulated cortices. Expression levels were weak in the basal ganglia, although the stria terminalis showed higher levels of Lgi1 expression (Figure 8A.1). Thalamic and hypothalamic areas also showed very weak to absent expression, even though low-level expression was detected around the third ventricle, which represents the medial habenular nuclear. The hippocampus now showed weak expression in CA1 and CA3 areas, although moderate expression was retained in the dentate gyrus as well as in the inferior tectal neuroepithelium and superior cerebellar peduncle (Figures 8A.2 and 8A.3).

Within the cerebral cortex at stage P5, moderate expression levels were seen in layers II/III and V of the neocortex. Expression in the hippocampus was maintained unchanged at this stage, with moderate levels seen in the dentate gyrus. We also observed Lgi1 expressing cells in the striatum (caudate/putamen), as shown in figures 8B.1 and 8B.2. Within the thalamus, Lgi1 protein levels were still weak, although the paraventricular thalamic region showed strong expression at this stage (Figure 8C.1). In the midbrain, expression was seen in the pretectal and tectal regions, tegmentum, and both superior and inferior colliculi, which became more diffuse at this stage. Within the pons, cerebellum and medulla oblongata the expression was now either weak or absent. The choroid plexus, however, still shows high levels of Lgi1 expression (Figure 8B.1).

### Lgi1 expression in the later postnatal stages of the murine brain

**Figure 9:**
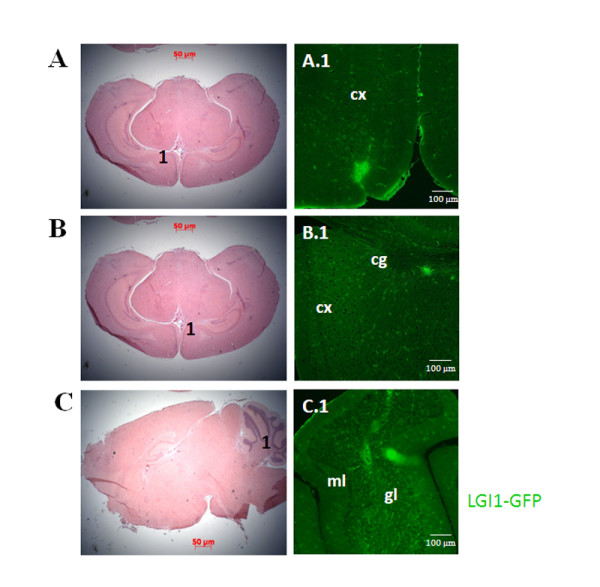
**Coronal and sagittal views of Lgi1 expression at (A) P10, (B) P15 and (C) P20 mouse brains**. **A**. Coronal section of the brain stained with hematoxylin and eosin used as reference to figure A.1. **A.1**. Shows Lgi1 expressing cells in the cortex (cx) at P10. **B**. Coronal section of the brain stained with hematoxylin and eosin used as reference to figure B.1. **B.1**. Shows Lgi1 distribution in the cortex (cx) and cingulum (cg) at P15. **C**. Sagittal section of the brain stained with hematoxylin and eosin used as reference to figure C.1. **C.1**. Shows Lgi1 expression in the granular cell layer (gl) of the cerebellar cortex at P20 but weak or no expression was seen in the molecular layer (ml). Scale bars = 100 μm

In later postnatal stages (P10, P15, and P20), Lgi1 expression levels were essentially the same (Figure 1), with strong expression in the choroid plexus, but in regions such as the neocortex, hypothalamus, midbrain, hippocampus, and cerebellum, expression was greatly reduced compared with embryonic stages described above (Figure 9). Expression in the medulla oblongata was absent compared to earlier embryonic stages where its expression was strong. The cerebral cortex now generally showed weak Lgi1 expression, although Lgi1 expressing cells were seen in areas of the frontal cortex (Figure 9A.1) and cingulum (Figure 9B.1). The globus pallidus showed little or no expression, while the striatum (caudate/putamen) showed relatively low levels of Lgi1 expression. Within the hippocampus, an area typically showing high levels of Lgi1 in the dentate gyrus, lower expression levels were observed throughout the region at these stages. Both the hypothalamus and thalamus showed little or no Lgi1 expression. Sagittal sections through the brain at P5 and P10 demonstrated that, although the thalamus expressed Lgi1 weakly overall, the paraventricular thalamic region appeared to have stronger Lgi1 expression (Figure 8C.1). In the dorsal midbrain, the pretectal nuclei showed weak expression as did the periaqueductal gray, an area surrounding the cerebral aqueduct, which showed moderate expression. More posteriorly, juxtaposed to the unstained superior colliculi, the inferior colliculi showed moderate levels of Lgi1 expression. In these stages, the pons and medulla oblongata did not express Lgi1. The cerebellum, although nearly completely devoid of Lgi1 expression, showed scattered staining in the Purkinje cells as we described previously [8]. Figure 9C.1 shows Lgi1 expression in the granular cell layer of the cerebellar cortex in P20 brain. Weak or no Lgi1 signal was seen in the molecular layer of the cerebellar cortex. The choroid plexus continued to show high expression levels at these stages.

### Co-expression of Lgi1 with Nestin, Doublecortin, and Beta-III Tubulin

**Figure 10:**
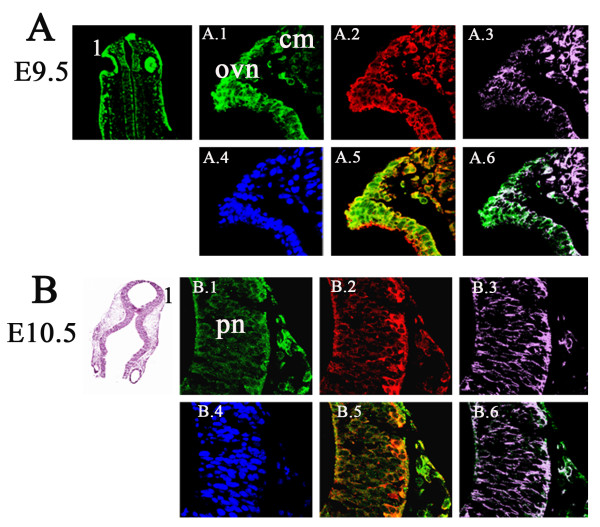
**Laser scanning confocal images showing co-expression of Lgi1-containg cells with nestin (n) and doublecortin (DCX) in distinct regions of mouse brain at embryonic stages E9.5 to E15.5**. **A**. Coronal view of the cephalic region at E9.5 showing regions of Lgi1 expression seen in Figure A.1-6. **A.1 **High magnification of the optic vesicle neuroepithelium (ovn) and cephalic mesenchyme (cm) showing Lgi1 expressing cells which also express DCX **A.2 **and nestin **A.3** overlaid in **A.5**. Lgi1 expressing cells do not express nestin as showed in Figure **A.6**. **A.4**. Shows DAPI staining for the same region. **B**. Frontal view (H & E stained) of an E10.5 mouse brain defining regions seen in Figures B.1-6. **B.1**. Shows Lgi1 expressing cells in pretectal neuroepithelium (pn) which also express DCX **B.2** and nestin **B.3** overlaid with DCX **B.5 **and nestin **B.6**. DAPI staining for this region is shown in **B.4**.

**Figure 11:**
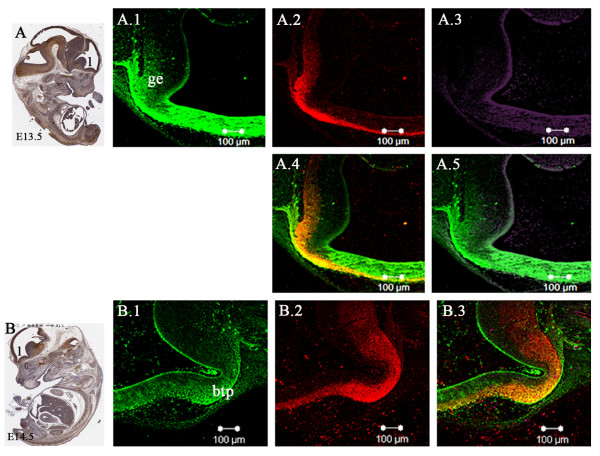
**Coexpression of neuronal precursor-specific markers with Lgi1 during early development of the brain. A**. Sagittal view (anti-GFP-peroxidase stained) of the E13.5 whole mouse embryo identifying regions shown in Figures A.1-5. **A.1**. Sagittal section through the ganglionic eminence (ge) showing Lgi1 expressing cells which also express DCX **A.2** and nestin **A.3**, overlaid of in A.4 and A.5, respectively. **B**. Sagittal view (anti-GFP-peroxidase stained) of E14.5 whole embryo identifying regions seen in Figures B.1-3. **B.1**. Shows Lgi1 expression in the basal telencephalic plate (btl) compared with DCX expression **B.2 **which are overlaid in **B.3**. Scale bar = 100 μm.

**Figure 12:**
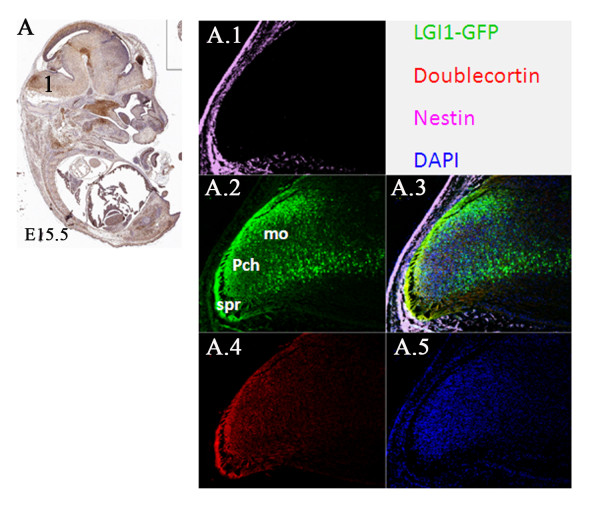
**Co-expression of neuronal precursor genes in the medulla oblongata of E15.5 embryos. A**. Sagittal view (anti-GFP-peroxidase stained) of the E15.5 whole mouse embryo showing regions described in Figures A.1-5. Nestin expression is identified in the **A.1 **subpial region (spr) and **A.2 **throughout the medulla oblongata (mo). **A.4 **shows DCX expression specifically in the parenchyma (pch) and subpial region (spr) which are overlaid in **A.3**, compared with DAPI staining **A.5**. Scale bars = 100 μm.

**Figure 13:**
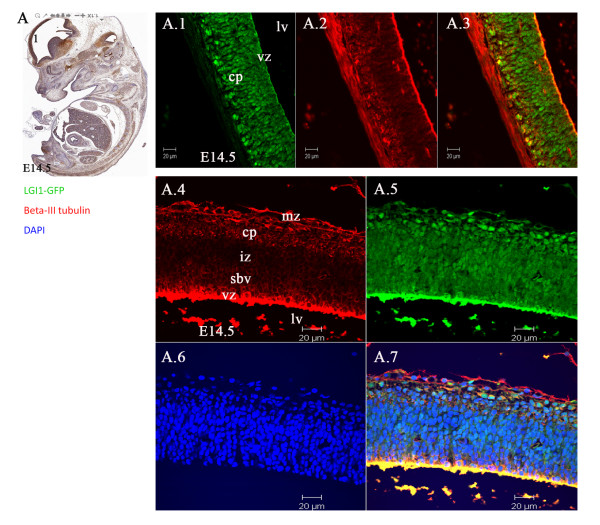
**Laser scanning confocal images showing co-expression of Lgi1 and beta-III tubulin in the cortical plate and ventricular zone of mouse brain at embryonic stage E14.5**. **A**. Sagittal view (anti-GFP-peroxidase stained) of whole embryo indicating regions shown in Figures A.1-7. **A.1**. Shows Lgi1 expressing cells through the ventricular zone (vz) and cortical plate (cp). **A.2**. Shows beta-III tubulin expression in the cortical plate, overlaid in **A.3 **and shown in higher magnification in **A.4 **and **A.5 **respectively and contrasted in DAPI in A.6. **A.7**. Shows Lgi1 expressing cells overlaid with beta-III tubulin in the marginal zone (mz), cortical plate (cp), and ventricular zone (vz). Beta-III tubulin is not expressed in intermediate zone (iz). Lgi1 signal is seen in intermediate (iz) and subventricular (sbv) zones. Lateral ventricle (lv). Scale bars = 20 μm.

In the developing brain at embryonic stages E9.5 and E10.5, co-expression of Lgi1 (Figure 10) was seen with nestin (neural progenitor cell marker), DCX (migrating neuroblast marker), and beta-III tubulin (neuron-specific marker) in neural structures such as the optic vesicle epithelium (Figure 10A), cephalic mesenchyme (Figure 10A), and pretectal neuroepithelium (Figure 10B). As the brain developed (E11.5-E15.5), co-expression of Lgi1 and nestin/DCX was observed in the ganglionic eminence (Figure 11A), basal telencephalic plate (Figure 11B), and medulla oblongata (Figure 12). At embryonic stage E15.5, co-expression of Lgi1, DCX and nestin was seen within the subpial and parenchyma layers of the medulla oblongata (Figure 12). This Nestin/DCX/Lgi1 co-expression is typical of migrating neuroblasts. Beta-III Tubulin expression was found for the first time in the cortical plate at embryonic stage E14.5 (Figure 13) and overlapped with Lgi1-expressing cells. Beta-III Tubulin has been suggested to be one of the earliest markers to signal neuronal commitment in primitive neuroepithelium. Co-expression of Slug (neural crest precursor cell marker prior to migration), RC2 (radial glial cell marker), NG2 (oligodendrocyte progenitor cell marker), and MAP2 (mature neurons) was not found in Lgi1-expressing cells at the time points used in this study.

## Discussion

The underlying mechanism behind the genetic predisposition to epilepsy conferred by mutations in the Lgi1 gene is still poorly understood. Although evidence derived from focused electrophysiological studies implicate abnormalities in synaptic transmission leading to hyperexcitability [16,17,29], there have also been suggestions that Lgi1 may also have a fundamental role in brain development [30], which may contribute to the seizure phenotype. In a BAC transgenic model expressing a truncated Lgi1 protein, for example, mutant Lgi1 led to neuronal restructuring involving inhibition of dendritic pruning and an increase in spine density [29]. Gross anatomical analysis of the brain in Lgi1 mutant null mice [16,18] however, did not reveal any major structural defects, although subtle changes could not be ruled out at this level of analysis. In humans, although imaging studies have been inconsistent [4,22-24], there are suggestions of abnormalities and reduced focal brain mass in ADTLE patients. Also, in our zebrafish studies, knockdown of Lgi1 in embryos resulted in increased apoptosis in subregions of the brain, leading to an overall reduction in brain size [31]. With the possibility that Lgi1 may play a role in overall brain development, and neuronal cell positioning in particular, it became important to define the structures in the developing brain that express Lgi1, since these are the cell lineages that are potentially most affected by loss of function. In this study we have broadly defined the temporal and spatial expression pattern in the developing brain and have demonstrated that, unlike the more restricted pattern seen in the early postnatal and adult mouse brain, expression of Lgi1 is widespread and highly orchestrated in a variety of cell types and is not restricted to differentiated neurons.

Evidence from our previous in vitro gene expression studies suggested that Lgi1 may have a role in axon guidance and cell migration [32]. Consistent with this suggestion, high levels of Lgi1 expression were found in regions of the developing brain such as the ganglionic eminence (GE), medulla oblongata (MO), ventricular zone (VZ) and telencephalon where mitotic neuronal precursors are located, and from which they migrate to populate the cortex and other areas. Lgi1 expressing cells in the pretectal area, GE, MO and telencephalon showed coexpression of Lgi1 with DCX (a marker for migrating neurons) and nestin (a marker for early stage neural progenitor cells). Nestin is expressed transiently during development and does not persist through adulthood, except in neuroprogenitor cells of the subventricular zone [33,34] which still showed Lgi1 expression in E18.5 embryos. Interestingly, the presence of cells co-expressing all three genes in this area suggests that these cells are migrating immature neurons. Also, the presence of oriented GFP-positive fibers in the subpial and parenchyma layers of the medulla oblongata suggest that Lgi1 may be involved in the relocation of immature neurons in the mouse subpial medullary region and may play a role in the migration of medullary precerebellar neurons in the caudal medulla [35].

Evidence from studies in adult brain [25,36], suggest that expression of Lgi1 is largely restricted to the hippocampus and cortical neurons. However, in our study we have shown that Lgi1 expression can be detected in the cephalic neuroepithelium at E11.5, which coincides with the first signs of neurogenesis in the mouse [37]. The appearance of Lgi1 expression in the cerebral cortex begins at ~E13.5, and peaks at E18.5. As development progresses, expression levels in the cortex progressively diminish. On the other hand, Lgi1 appears not to be involved in early differentiation and maturation of glial lineage since Lgi1-expressing cells do not coexpress RC2 (radial glial cells) or NG2 (oligodendrocyte precursor marker). Even thought differentiated neurons expressed Lgi1, many of these cells do not express MAP2 at the observed stages. We speculate that once these cells have migrated to their final destinations, Lgi1 expression levels decay.

Lgi1 was also found to be strongly expressed in the basal telencephalic plates that will develop into the basal ganglia associated with motor functions. DCX-expressing cells in the ventral portion of the basal telencephalic plates also express Lgi1, suggesting that these DCX-Lgi1-expressing cells migrate and will populate cortical and subcortical regions. In addition, Marín et al [38] argue that patterning of the basal telencepahlon is crucial for the growth of cortical axons as well as for guiding cortical projections involved in sensory-motor information. The presence of Lgi1 in migrating neuroblasts is consistent with a function in cortical and medullar axon guidance, as suggested by our gene expression studies in model cell systems [32]. Beta-III tubulin is suggested to be one of the earliest markers to signal neuronal commitment in primitive neuroepithelium, and it is accepted as a neuron-specific protein marker, which is highly expressed in cortex at birth and then its expression levels decrease with increasing postnatal development [39]. During brain development, the maturation of neuroblasts into neurons is accompanied by up-regulation of beta-III tubulin. Coexpression of Lgi1 and beta-III tubulin was detected in the ventricular and subventricular zones, as well as the cortical plate, suggesting a conserved neurogenic program in these cells from embryonic stages to adult life, which further implies that Lgi1 may participate in normal neuronal development.

We also observed Lgi1 expression in many areas of the developing sensory system, for example, the tongue (taste buds), eye, and olfactory bulb. The olfactory system is anatomically connected to the amygdala, lateral septum, hypothalamus and the hippocampus, which are part of the limbic system [40] and which also express Lgi1. This correlation is consistent with a role for Lgi1 in the development of limbic encephalitis [5]. Within the hippocampus, expression levels are found primarily in the dentate gyrus and scarcely within the CA1-CA3 region of the pyramidal cell layer. There is evidence that Lgi1 also regulates intra-hippocampal circuit formation and the dentate gyrus plays a critical role in seizures, since it appears to have low epileptogenic thresholds [29]. Although thalamic protein levels are weak overall, the paraventricular thalamic area expresses Lgi1. According to Huang et al [41], the thalamic paraventricular nucleus receives the highest innervation from hypothalamic hypocretin/orexin neurons, generating substantial excitation of the medial prefrontal cortex. Major inputs into the anterior thalamic group are from the hypothalamus, the hippocampus, and the tectum while output from this region is mainly to the cingulate gyrus. In the developing mouse eye, Lgi1 expression was seen in the optic vesicle, optic stalk and optic cup, as well as the neural retina and the retinal pigment epithelium. Lgi1 expression is seen in the retinal ganglion layer, and dominant-negative mutations in transgenic mice lead to abnormal postnatal pruning of retinal axons in the visual relay thalamus [42]. In zebrafish studies, Lgi1 expression was seen in the epithelium surrounding the lumen of the optic vesicle [43] and interestingly, in early zebrafish embryos, loss of Lgi1 results in abnormal eye development [31], supporting its critical role in development of this organ.

## Conclusions

Several general patterns of Lgi1 distribution were apparent during embryonic brain development. Lgi1 expression appears to spread throughout the dorsal-most brain structures with only weak levels in the ventral regions in early embryo stages within the medulla oblongata showing the strongest expression. This dorsoventral differentiation also appears in the hippocampus, where the dentate gyrus showed more robust expression. Lgi1 expression also appears to follow a superficial to deep, as well as a caudal to cephalic axis. At E10.5, the strongest signal was found in the surface ectoderm, as well as the infundibular recess of the diencephalon and dorsal midbrain in the pretectal neuroepithelium. As development progresses, Lgi1 expression moves interiorly into the neocortex and other more caudal structures, expression in the pretectum and diencephalon areas begins to subside, as shown by weak expression in basal ganglia and thalamic nuclei, although expression in the paraventricular thalamic nucleus remains strong at P5. Overall, the spatial and temporal expression profile for Lgi1 during embryogenesis and postnatal stages is consistent with the idea that it plays an important role in normal brain development, especially neuronal migration and synapse formation.

## Methods

### Lgi1-EGFP mice

The generation of the LGI1-EGFP BAC transgenic mice has been reported previously [8]. Mice were housed under standard conditions and treated in accordance with the Guidelines for the Care and Use of Laboratory Animals of Medical College of Georgia.

### RT-PCR

Mouse brains were isolated and immediately frozen in liquid nitrogen. Using a polytron homogenizer, whole embryos or brains were then homogenized in trizol (Invitrogen) and RNA was isolated as recommended by the manufacturer. Lgi1 was amplified using 5'-GATCCATTCCACGCACCGTTCCTC3' (Forward) and 5'TCTTCTCTACGTGGTCCCATTCCA-3' (Reverse) primer sequences and resolved in 1% agarose gels.

### Histology and confocal microscopy

Embryos were removed from pregnant female mice and fixed in a 4% paraformaldehyde solution in PBS for at least 24 hours. Embryos were then first dehydrated in stepped concentrations of ethanol solution from 70% to 100% and then placed in xylene before being embedded in paraffin. Brains from neonates were dissected and quickly fixed in 4% paraformaldehyde solution and similarly mounted in paraffin. 7 μm coronal and sagittal sections were collected onto plus-charged glass slides (Fisher Scientific, Pittsburgh, PA). After deparaffinization in xylene, and rehydration, the tissues were permeabilized with 0.1% triton X-100 in PBS. Processed sections were then reacted with specific primary and secondary antibodies (see below). Brightfield and confocal images were obtained using a Zeiss microscope and LSM500 software. Several different reference resources were used as an aid in identification of anatomical structures [44-48].

### Antibodies

Immunogen affinity-purified polyclonal anti-EGFP antibody (Abcam, ab6556) was used for confocal analysis. Anti-NG2, anti-Slug, and Doublecortin (sc-18) antibodies were obtained from Santa Cruz Biotechnology, Inc. Anti-Nestin (MAB353) and Anti-Beta III Tubulin (MAB5564) monoclonal antibodies were obtained from Millipore Corporation. Anti-MAP2 was from Cell Signaling, and Anti-RC2 was from UC Davis. The secondary antibodies used for primary antibody detection were peroxidase conjugated anti-rabbit, anti-goat, and anti-mouse IgG, all obtained from Jackson ImmunoResearch Laboratories.

## Authors' contributions

JS performed the preparation of the embryos and confocal imaging and co-wrote the manuscript. JKC provided expertise in the analysis of the BAC transgenic mouse system and co-wrote the manuscript. GW provided invaluable assistance in the characterization of the embryonic regions expressing Lgi1 and advice about the interpretation of the co-localization studies. All authors have read and approve the manuscript.
